# The Emerging Role of Myeloid-Derived Suppressor Cells in Tuberculosis

**DOI:** 10.3389/fimmu.2019.00917

**Published:** 2019-04-30

**Authors:** Tandeka Magcwebeba, Anca Dorhoi, Nelita du Plessis

**Affiliations:** ^1^Division of Molecular Biology and Human Genetics, Department of Biomedical Sciences, Faculty of Medicine and Health Sciences, South African MRC Centre for Tuberculosis Research, DST and NRF Centre of Excellence for Biomedical TB Research, Stellenbosch University, Stellenbosch, South Africa; ^2^Institute of Immunology, Friedrich-Loeffler-Institut, Greifswald, Germany; ^3^Faculty of Mathematics and Natural Sciences, University of Greifswald, Greifswald, Germany; ^4^Department of Immunology, Max Planck Institute for Infection Biology, Berlin, Germany

**Keywords:** myeloid-derived suppressor cells, *Mycobacterium tuberculosis*, infectious disease, immunosuppression, innate immunity

## Abstract

Myeloid cells are crucial for the host control of a *Mycobacterium tuberculosis* (*M.tb*) infection, however the adverse role of specific myeloid subsets has increasingly been appreciated. The relevance of such cells in therapeutic strategies and predictive/prognostic algorithms is to promote interest in regulatory myeloid cells in tuberculosis (TB). Myeloid-derived suppressor cells (MDSC) are a heterogeneous collection of phagocytes comprised of monocytic- and polymorphonuclear cells that exhibit a potent suppression of innate- and adaptive immune responses. Accumulation of MDSC under pathological conditions associated with chronic inflammation, most notably cancer, has been well-described. Evidence supporting the involvement of MDSC in TB is increasing, yet their significance in this infection continues to be viewed with skepticism, primarily due to their complex nature and the lack of genetic evidence unequivocally discriminating these cells from other terminally differentiated myeloid populations. Here we highlight recent advances in MDSC characterization and summarize findings on the TB-induced hematopoietic shift associated with MDSC expansion. Lastly, the mechanisms of MDSC-mediated disease progression and future research avenues in the context of TB therapy and prophylaxis are discussed.

## Introduction

Tuberculosis (TB) remains a leading cause of global mortality (1, 2). Insufficient understanding of TB disease mechanisms represents a major factor impeding its elimination (3). A recent paradigm describes TB as a continuous spectrum of processes, rather than a binary distribution between asymptomatic latent infection and active disease (3–6). This underscores the complex pathophysiology of TB, including multiple cellular effectors, regulators, and checkpoints. Myeloid cells, including neutrophils and monocytes, function both as initial effectors and during the lag phase of T-cell responses to restrict *M.tb* burden and limit disease progression by activating pro-inflammatory signaling pathways, recruiting additional phagocytes, ingesting bacilli, up-regulating bactericidal mechanisms and inducing antigen-specific adaptive immunity (7–9). Even so, myeloid cells can switch from facilitating protective immunity, to aiding pathological processes, by enhancing TB progression via immunosuppression and dysregulated inflammation (8). Chronic mycobacterial infection triggers the generation of immunosuppressive/tolerogenic myeloid cells, which were initially referred to as “innate natural suppressor cells” (10–12). Subsequent studies have coined these as myeloid-derived suppressor cells (MDSC) (13, 14).

## Mycobacteria-Induced Natural Suppressor Cells

Early reports on regulatory myeloid cells in mycobacterial infection came from *in vivo* and *in vitro* studies with *Mycobacterium bovis* Bacillus Calmette-Guerin (BCG) (11, 15–17).In these studies it was indicated that, systemic delivery of mycobacteria induce expansion of hematopoietic progenitor cells in the bone marrow, with the subsequent migration of these cells to the peritoneal cavity and their activation in the spleen (11, 18). It was further reported that BCG could induce the expansion of bone-marrow derived and splenic natural suppressor cells and that these cells could inhibit cell-mediated immunity, notably by suppressing the migratory capacity and proliferation of helper and cytotoxic T-cells (15, 16). T-cell immunosuppression was attributed to the presence of macrophage-like natural suppressor cells, the production of high levels of IL-1 and soluble suppressive factors (16, 19). Natural suppressor cells were later linked to MDSC. Natural suppressor cells from mice exposed to mycobacterial products in Complete Freud's adjuvant (CFA), shared similar phenotypic and functional features with MDSC (10). These cells highly expressed the markers of myeloid origin and differentiation, Gr-1 and CD11b, and inhibition of T-cell proliferation and IFN-γ production was linked to NO production in splenocytes (10). Subsequent studies validated the presence of MDSC during BCG infection (13) and in patients with active TB (14). Thus, initial observations of natural suppressor cells were during mycobacterial insult and established that the generation of these cells was driven by the mycobacterial products.

## MDSC Characterization in Mycobacterial Infections

Identification of MDSC requires a combination of assays comprising of immunophenotyping, enzyme measurements, and suppressive tests (20). Markers employed for detection of human MDSC allow, to some extent, their differentiation from monocytes and neutrophils, although this is cumbersome in mice (21). At present, three commonly reported MDSC subsets identified in human TB include early stage MDSC (e-MDSC), polymorphonuclear-MDSC (PMN-MDSC), and monocytic-MDSC (M-MDSC) (14, 22, 23). Immunosuppressive eosinophilic MDSC have recently been described during chronic *Staphylococcus aureus* infection *in vivo* but require validation in other diseases (24). MDSC enriched in TB patients, according to recent recommendations using a ficoll density-gradient (22, 23), have been classified as e-MDSC (LIN1^−^HLA-DR^−/lo^CD11b^+^CD33^+^), PMN-MDSC (HLA-DR^−/lo^CD11b^+^CD14^−^CD15^+^CD33^+/dim^) and M-MDSC (HLA-DR^−/low^CD11b^+^CD14^+^CD15^−^CD33^+^) (20). Instead of a specific subset, M-MDSC population has been described as a heterogenous population of cells, in different maturation stages (20). Since there are no specific markers for MDSC, ambiguity with other myeloid cells that have similar phenotypic characteristics and functional properties exists, especially after pathogen exposure. For instance, infection of monocytes with *Candida albicans* fungal cells and exposure to fungal components subverts monocyte differentiation to immunosuppressive dendritic cells. The phenotype of the subverted DC is characterized by the expression of CD14 with a lack of CD1a molecule, presence of CD83 and CD86 but a relatively low expression of MHC class II and CD80. These cells produce IL-12 but are associated with the release of IL-10 and IL-6 (25). Similarly our group has demonstrated that CD14+ M-MDSC production of IL-10 and IL-6 is associated with either absent, or relatively low levels of HLA-DR and CD80 (14, 26). Thus, an unequivocal marker that is able to distinguish myeloid cell population and subsets in biological samples such as whole blood culture and tissue is required. Whilst there is no specific marker for M-MDSC yet, utilization of LOX-1 as a unique PMN-MDSC marker has been proposed but (27) requires validation in TB patients.

In murine TB, PMN-MDSC are phenotypically Gr-1+CD11b^+^Ly6G^+^Ly6C^lo^/^int^ and M-MDSC Gr-1+CD11b^+^Ly6G^−/l0^Ly6C^hi^, yet functional assays are essential for their classification (28–30).

Morphological characterization has been used as a confirmatory tool to distinguish MDSC from other myeloid cells in TB samples (22, 28). Immature myeloid cells identified as PMN-MDSC share similar morphological characteristics with neutrophils, as they show ring-shaped or band nuclei. This nuclear shape can, however, be present in neutrophil progenitors and young neutrophils. Utilization of CD10 for human specimens (21) along with suppressive assays may help distinguish PMN-MDSC from non-suppressive immature neutrophils. MDSC likely encompass cells at different maturation stages with a distinct activation status and functional role. For instance, expansion of MDSC with the phenotype Lin^−/l0^HLA-DR^−/lo^CD11b^+^CD14^+^CD33^+^CD80^+^, was described in patients with active TB and their frequency correlated with disease progression (14). CD80 up-regulation upon successful TB chemotherapy was associated with MDSC differentiation into macrophages and dendritic cells (14). In mice, accumulation of an immature, heterogeneous population of Gr1^dim^CD11b^+^ cells with un-segmented nuclei, which also expresses progenitor markers (CD117^+^CD135^+^), was observed during the advanced disease in TB prone animals (28).

## Tissue Compartmentalization and Dynamics of MDSC in TB

In murine models MDSC were detected in the blood during BCG vaccination (13). In adults and children suffering from TB, MDSC frequencies in the periphery were comparable to those found in cancer patients (14). All MDSC subsets have been identified in the blood of TB patients, yet relative ratios, within different biological samples/fluids, differ in various studies (14, 22, 23). For instance, PMN-MDSC are enriched in the lung, specifically in bronchoalveolar lavage (BAL) samples of pulmonary TB patients (22) whilst the prevalence of a M-MDSC subset has been described in pleural effusions (14). Compartmentalization of the different MDSC subsets during TB in humans may be site-specific and likely dependent on the disease stage. Such an assumption is supported by findings from experimental TB. In naïve mice, MDSC can be detected at very low frequencies primarily in bone marrow. During acute TB, MDSC mildly accumulates in the lung and upon disease progression their numbers dramatically increase in all aforementioned organs and are also detected in the blood (28). High levels of MDSC in bone-marrow suggests that their genesis occurs primarily via medullary hematopoiesis. A pro-inflammatory environment, abundant in IL-6/G-CSF/PROK-2 may promote myelo- and granulopoiesis, whereas recruitment of MDSC to the lung could be directed by abundant S100-proteins/MMP-9/G-CSF (20, 29). Accumulation of MDSC in the lung parenchyma parallels TB progression in susceptible mice (29, 30). In *M.tb*-infected-necrosis prone mice, M-MDSC accumulate at the edges of necrotic granulomas (30). A recent study further strengthened the case for MDSC as regulators of granuloma biology. Human *ex vivo* generated M-MDSC promote mycobacterial replication in *in vitro* established granulomas, in a process dependent on abundant release of IL-10 (26).

Dynamics of MDSC subsets through-out the course of the TB disease spectrum (31) are relevant for disease pathophysiology. In TB patients, MDSC abundances have not yet been clearly linked with the extent of disease, e.g., by establishing a correlation between their frequencies and lung radiological involvement, smear grading or bacterial burden. Community controls from a high-exposure region and also individuals with remote exposure to *M.tb*, display very low levels of circulating MDSC, yet frequencies of MDSC increase in recently exposed house hold contacts (HHC) of TB patients (19). MDSC presumably emerge in incipient TB with their increased frequency associated with disease progression. TB-resistant mice that are devoid of necrotic granulomas have minimal levels of MDSC, whilst necrotic prone mouse strains NOS2^−/−^ (knock-out), C3HeB/FeJ, 129S2 (immunocompetent) exhibit higher frequencies with the highest levels observed in immunodeficient (RAG2^−/−^) animals (29, 30). The accumulation of MDSC in necrotic granulomas has been associated with the inability to control *M.tb* infection and lung pathology (28, 29). Pulmonary tuberculosis manifests differently than pleural tuberculosis and MDSC biology in pleural cavities still needs further characterization. In TB patients, MDSC are present in pleural effusions and blood and the immunosuppressive potential of MDSC from individuals with a long term infection exceeds the suppression of cells isolated from people with recent *M.tb* exposure, which also affects CD8 T-cell responsiveness (14). Upon a successful cure, MDSC frequencies decrease to levels observed in healthy controls (14). In children, completion of standard TB treatment was not accompanied by a MDSC decline, likely reflecting the more complex disease presentation of pediatric TB and possibly the polarization of the immune response which may be different to adult immune response (32).

## MDSC Directly Interact With Mycobacteria

Lung-residing M-MDSC harbor *M.tb* and promote bacterial growth through mechanisms involving IL-4/IL4Rα signaling (29). Despite the production of nitric oxide (NO), a potent anti-mycobacterial molecule, MDSC are inefficient at controlling mycobacterial growth (13). Although *ex vivo* generated human MDSC are not able to provide a niche for fast replication of *M.tb* when compared to macrophages, they do however exert a potent suppressive activity against T-cells upon infection (26).Recent reports indicate that myeloid cell ontogeny affects their capacity to support mycobacterial growth. Interstitial macrophages, supposedly originating from circulating monocytes, allow lower *M.tb*. replication rates as compared to fetal germline derived alveolar macrophages (AM). This phenomenon has been linked to the dramatically different metabolic states of AM and interstitial macrophages, with highly up-regulated fatty acid uptake and β-oxidation vs. high glycolytic activity, respectively (33). Pre-existing metabolic bias of myeloid cells controls *M.tb* growth (33). Of note, tumor-infiltrating MDSC preferentially use fatty acid-β-oxidation (FAO) as a primary energy source, display up-regulation in FAO genes and increases the oxygen consumption rate (34). We, and others have previously shown that MDSC are capable of mycobacterial internalization, however, they display poor microbicidal activity (13, 26). Considering that *M.tb* uses host fatty acids and cholesterol, the metabolic status of MDSC likely offers a nutritional niche supporting *M.tb* maintenance (35, 36). Whether FAO affects *M.tb* survival within MDSC remains to be validated. In the same vein, the metabolic state of *M.tb* as well as its subcellular localization within MDSC are largely unknown and should be defined.

## Mediators of MDSC Expansion and Activation in TB

**Figure 1:**
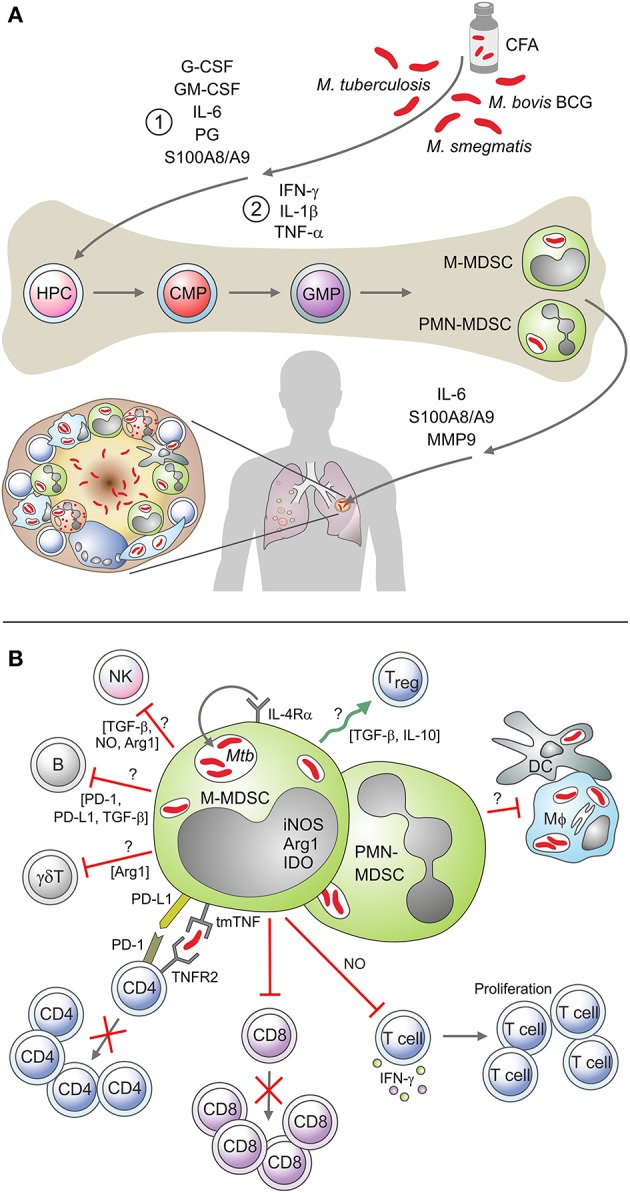
**(A)** Mediators of MDSC expansion and activation in a mycobacterial environment. A schematic depicting mediators associated with the proposed “two-signal” MDSC expansion and activation process, in a mycobacterial environment. These include cytokines, chemokines, calcium-binding proteins, and matrix metalloproteinases. **(B)** MDSC cellular interaction and mediators of immunosuppression in a mycobacterial environment. Examples of the known and suggested interactions of MDSC with other immune cells in the mycobacterial setting, including the soluble mediators associated with their immunosuppressive activity.

Expansion and activation of MDSC is mediated by chronic, low-grade inflammation, resulting in the pathological activation of myeloid cells (37). Currently, it is difficult to discriminate signals mediating MDSC expansion from those mediating MDSC activation. Recent findings support a two-step process involving cellular expansion, licensing, and activation (37, 38). First, chronic exposure to GM-CSF, IL-6, prostaglandins, and alarmins such as S100A8/9 (38, 39) promote “emergency myelopoiesis,” impede on terminal maturation of myeloid progenitors. The second phase involves activation of these “licensed” myeloid cells, through the panoply of inflammatory cytokines (e.g., IFN-γ, IL-1β, IL- 6, TNF-α, IL-4), DAMPs (e.g., HMGB1), and likely also PAMPs (e.g., LPS) to obtain suppressive functions (37–39). Such factors are produced during TB and enriched in TB-susceptible mice accumulating MDSC (Figure 1A) (29). Additional molecules detected in TB lesions, including prokineticin 2 (PROK 2) and MMP9, which promote MDSC accumulation in target organs, may also regulate MDSC expansion (29). Recent reports indicate that transmembrane TNF-alpha regulates the activation and expansion of PMN-MDSC and M-MDSC in the pleural cavity of BCG infected mice (40). In mycobacterial infections, M-MDSC are induced regardless of key virulence factors, as *M.tb, M.smeg*, and BCG have proven to induce MDSC (13). Consequently, due their immunosuppressive activity and high frequency during disease progression, MDSC have been identified as one of the factors that may contribute to a low BCG vaccine efficacy (41). Other factors may include geographical location, helminthic co-infection, route of BCG administration and mycobacterial strain (42). It is important to note that the robust cytokine response often observed following BCG vaccination, contradicts the MDSC functions described above. We suspect that this perceived discrepancy, could be ascribed to the requirement of a 2nd activation signal or the mycobacterial strain-specific differences on MDSC function. Alternatively, the MDSC suppressive function might stretch beyond T-cell immunity and affect other cell subsets which are rarely evaluated following BCG vaccination, with the route of the vaccination and the age of the vaccine, also contributing to the outcome. The role of live bacteria in regions from which MDSC originate, such as immature bone marrow cells, still need to be investigated.

Mycobacterial glycolipids contained in CFA promote the expansion of the MDSC (10). A comprehensive comparison of “licensed” monocytes, M-MDSC and additional monocytic subsets present in the *M.tb* infected lung is necessary to distinguish pathways driving MDSC genesis. Advanced techniques such as quantitative shotgun proteomics, RNASeq and chromatin ATAC mapping should provide insights into potentially discriminating markers and differentiation pathways.

## MDSC Immunosuppressive Mechanisms During TB Infection

MDSC exert their immunosuppressive activity through mechanisms that involve soluble factors, cell membrane molecules and the modulation of local concentrations off of metabolites and amino acid (20, 43). Most studies focus on T-cell suppression (ref), however MDSC also interact with macrophages and dendritic cells, and induce regulatory B- and T-cells (44–46). Such interactions have not yet been considered in TB (Figure 1B). The interaction of MDSC with T-cells has been established in TB patients, though the effects on antigen-specific responder lymphocytes still await clarification. Suppression of polyclonal stimulated CD4 and CD8 T-cells involves the inhibition of cytokine production, T-cell activation and modulation of T-cell trafficking (14). Whereas, PMN-MDSC expansion correlates with abundant plasma NO (22), phenotypically resembling MDSC present abundant indoleamine 2,3-dioxygenase (IDO) and arginase-1 (ARG-1) (32). In BCG vaccinated mice, iNOS-mediated tendency of MDSC to dampen T-cell priming, suppress polyclonal T-cell proliferation and IFN-γ release (13). iNOS mediates the suppression of lymphocytes also in murine TB, though *in situ* co-expression of ARG1 and iNOS has been detected in lung lesions (29).Cell surface molecules involved in the regulation of MDSC functions have been identified in experimental TB studies. In mice with mycobacterial pleurisy, tmTNF-α regulates MDSC activity through the cell-to-cell interaction between tmTNF-α expressing MDSC and TNFR2 expressing CD4 T-cells (40). Human MDSC up-regulate PD-L1 upon *in vitro* mycobacterial infection (26) and employ this check-point molecule to restrict T-cell proliferation (26, 47). IFN-γ counteracts PD-L1 induced suppression (47) and this may explain the profound immunosuppression in end stage TB patients. Relevance of additional enzymes enriched in MDSC purified from cancer patients, such as NADPH and COX2 (20), as well as roles of autophagy molecules (48), remain to be established in TB. Of paramount importance will be the deciphering of interactions between MDSC and macrophages, as those cells harbor and aid restricting bacillary replication. The capacity of MDSC to modulate Treg dynamics, induce Breg and alter NK activity in TB is also unknown. High dimensional analyses, e.g., mass cytometry and histo-cytometry could establish effects on MDSC on various immune cells and facilitate the in-depth functional characterization of these cells. MDSC may further contribute to TB reactivation by exacerbating the immunosuppressive effects of immunotherapy such as anti-TNF agents, absence of TNF-alpha has been associated with an increased bacterial load and T-cell immunosuppression (49, 50).

## MDSC and TB Co-Morbidities

Diseases promoting TB development are typically linked to immunosuppression or dysregulation of immunity and encompass HIV (51, 52) and diabetes (53, 54). In addition, undernourishment, alcoholism, and smoking are considered risk factors for TB. Currently, the precise role of MDSC in these conditions and subsequent implications for TB are not clear. MDSC have been reported in HIV infection, but a prevalence of distinct subsets during co-infection has not been unanimously established. Some studies report high frequencies of the PMN-MDSC subset (52, 55–57) whilst others describe increased M-MDSC populations in AIDS patients (58–61). MDSC frequencies correlate with AIDS progression and viral load (51, 59), while anti-retroviral therapy (ART) reduces systemic MDSC frequencies (44, 62, 63). Even HIV exposed uninfected children display abundant circulating MDSC (32). MDSC activity in an HIV environment involves enhanced IL-10 production, induction of CD4+CD25+FoxP3+Tregs and suppression of T-cell responses, notably inhibition of IFN-gamma release by autologous T-cells (52, 60). Such effects may contribute to development of TB in LTBI people infected with HIV, however further studies are required to elucidate the precise role of HIV-induced MDSC in TB reactivation. Very few reports focus on MDSC in diabetes. Recent trials suggest a beneficial effect with MDSC protecting against the development of type-2 diabetes (T2DM) in humans (64). Interestingly, the anti-diabetic drug metformin, showing efficacy as an adjunct therapy in TB (65), causes reduction of MDSC in cancer patients (66). Metformin's effect on MDSC in TB patients has not been evaluated. Smoking is regarded as a predisposing factor that can accelerate TB progression. Although smoking has been associated with MDSC expansion and generation in COPD patients (67, 68), the role of these cells in TB is not clear and should be clarified. Obesity-driven chronic, low-grade inflammation and leptin interaction has also shown to induce MDSC that, although protective against some metabolic dysfunctions, appear to be detrimental to tumor progression (69). At the other end of the spectrum, malnutrition has also been correlated to MDSC induction, suggesting a link with diseases characterized by wasting and malnutrition, such as TB (70). It is tempting to speculate that enhanced MDSC levels in diseases and conditions causing alterations in immune reactivity may contribute to TB reactivation, however this remains to be tested.

## Therapeutic Strategies Targeting MDSC in TB

Shortly after identification of MDSC in TB patients and murine models, these cells emerged as promising targets for adjunct host-directed therapy (HDT) approaches (8, 41, 71). The focus of such strategies has been to reverse the impact of MDSC on T-cell immunity in TB by implementing host modulating therapeutic strategies such as those blocking MDSC induction or activation, inhibiting MDSC function or reversing their suppressive function. These strategies have been recently reviewed elsewhere (71). More recently, denileukin diftitox, an anti-neoplastic agent comprised of IL-2 and Diphtheria toxin, potentiates standard TB treatment in a mouse model through the elimination of MDSC and Treg (72). Similarly, combined immunotherapy consisting of ATRA and alpha galactosylceramide as an adjunct immunotherapy improved standard TB treatment (73). Other studies on ATRA have reported the reduction of MDSC and increase in T-cell number with an impact on bacillary loads and lung pathology (13, 29). Tasquinimod (TSQ), a quinoline-3-carboxyamide analog, targets S100A9, a molecule which has been implicated in MDSC accumulation and function. TSQ is in clinical development for the treatment of various cancers and has recently shown to significantly enhance the antitumor effects of immunotherapeutics in cancer mouse models, by inhibiting the suppressive function of MDSC and tumor-associated macrophages (TAM) (74). More recently, TSQ treatment in an acute mouse model of TB, enhanced *M.tb* clearance, reduced Treg and MDSC frequencies and enhanced the efficacy of the standard treatment regimen (75).

Cytokines indirectly affect MDSC accumulation/function and a recent study has shown that IFN-γ decreases the suppressive function of MDSC by reducing the arginase activity suppressing PD-1/PD-L1 (47). Although not yet tested in TB, a combination treatment of IL-17R and IFN-γ has shown potential in cancer, by reducing the levels of MDSC and increasing T-cells (76). Other MDSC targeting agents tested in cancer, which have shown potential in TB, but with unknown effects on MDSC, include metformin, tyrosine kinase inhibitors (imatinib), PDE-5 inhibitors, and arginase inhibitors (71). The COX-2 inhibitor, etoricoxib, is currently evaluated as HDT for TB and its effect on MDSC levels will be considered in the trial (NCT02503839).

## Conclusion

The MDSC arena has experienced several research advances in the context of infectious diseases. Nonetheless, the complex and protracted nature of *M.tb* infection along with challenges in biology of MDSC research have delayed comprehensive investigations on MDSC in the TB field. Ultimately, MDSC research in TB would be insignificant without an eventual tangible contribution to the clinical benefit of patients. Development of immunotherapies targeting MDSC is undergoing a slow but steady progress, however many TB HDT trials fail to consider the impact of these treatments on MDSC function and frequency. The lack of compounds targeting MDSC specifically, contributes to this problem. The safety, efficacy, dose, and timing of interventions targeting MDSC in TB, will also require careful evaluation, and so too will the effect of novel neonatal vaccines and adult re-vaccination strategies on MDSC genesis. Greater focus on these and other MDSC knowledge gaps is expected to accelerate the discovery of effective TB immunotherapies, thereby contributing to an increased TB cure rate, more durable clinical responses and superior control of drug-resistant *M.tb* strains.

## Author Contributions

All authors listed have made a substantial, direct and intellectual contribution to the work, and approved it for publication.

### Conflict of Interest Statement

The authors declare that the research was conducted in the absence of any commercial or financial relationships that could be construed as a potential conflict of interest.
