# Corrigendum: The Emerging Role of Myeloid-Derived Suppressor Cells in Tuberculosis

**DOI:** 10.3389/fimmu.2019.01528

**Published:** 2019-07-02

**Authors:** Tandeka Magcwebeba, Anca Dorhoi, Nelita du Plessis

**Affiliations:** ^1^Division of Molecular Biology and Human Genetics, Department of Biomedical Sciences, Faculty of Medicine and Health Sciences, South African MRC Centre for Tuberculosis Research, DST and NRF Centre of Excellence for Biomedical TB Research, Stellenbosch University, Stellenbosch, South Africa; ^2^Institute of Immunology, Friedrich-Loeffler-Institut, Greifswald, Germany; ^3^Faculty of Mathematics and Natural Sciences, University of Greifswald, Greifswald, Germany; ^4^Department of Immunology, Max Planck Institute for Infection Biology, Berlin, Germany

**Keywords:** myeloid-derived suppressor cells, *Mycobacterium tuberculosis*, infectious disease, immunosuppression, innate immunity

In the original article, reference “37” was incorrectly written as “Millrud CR, Bergenfelz C, Leandersson K. On the origin of myeloid-derived suppressor cells. *Oncotarget*. (2017) 8:3649–65. doi: 10.18632/oncotarget.12278” It should be “Ribechini E, Hutchinson JA, Hergovits S, Heuer M, Lucas J, Schleicher U, et al. Novel GM-CSF signals via IFN-γR/IRF-1 and AKT/mTOR license monocytes for suppressor function. *Blood Adv*. (2017) 1:947–60. doi: 10.1182/bloodadvances.2017006858.”

In the original article “Ribechini et al. 2017” was not cited in the article. The citation has now been inserted in the section **Mediators of MDSC expansion and activation in TB**, paragraph one, and should read:

“Expansion and activation of MDSC is mediated by chronic, low-grade inflammation, resulting in the pathological activation of myeloid cells (37). Currently, it is difficult to discriminate signals mediating MDSC expansion from those mediating MDSC activation. Recent findings support a two-step process involving cellular expansion, licensing, and activation (37, 38). First, chronic exposure to GM-CSF, IL-6, prostaglandins, and alarmins such as S100A8/9 (38, 39) promote “emergency myelopoiesis,” impede on terminal maturation of myeloid progenitors. The second phase involves activation of these “licensed” myeloid cells, through the panoply of inflammatory cytokines (e.g., IFN-γ, IL-1β, IL- 6, TNF-α, IL-4), DAMPs (e.g., HMGB1), and likely also PAMPs (e.g., LPS) to obtain suppressive functions (37–39). Such factors are produced during TB and enriched in TB-susceptible mice accumulating MDSC (Figure 1A) (29). Additional molecules detected in TB lesions, including prokineticin 2 (PROK 2) and MMP9, which promote MDSC accumulation in target organs, may also regulate MDSC expansion (29). Recent reports indicate that transmembrane TNF-alpha regulates the activation and expansion of PMN-MDSC and M-MDSC in the pleural cavity of BCG infected mice (40). In mycobacterial infections, M-MDSC are induced regardless of key virulence factors, as *M.tb, M.smeg*, and BCG have proven to induce MDSC (13). Consequently, due their immunosuppressive activity and high frequency during disease progression, MDSC have been identified as one of the factors that may contribute to a low BCG vaccine efficacy (41). Other factors may include geographical location, helminthic co-infection, route of BCG administration and mycobacterial strain (42). It is important to note that the robust cytokine response often observed following BCG vaccination, contradicts the MDSC functions described above. We suspect that this perceived discrepancy, could be ascribed to the requirement of a 2nd activation signal or the mycobacterial strain-specific differences on MDSC function. Alternatively, the MDSC suppressive function might stretch beyond T-cell immunity and affect other cell subsets which are rarely evaluated following BCG vaccination, with the route of the vaccination and the age of the vaccine, also contributing to the outcome. The role of live bacteria in regions from which MDSC originate, such as immature bone marrow cells, still need to be investigated.”

The authors apologize for this error and state that this does not change the scientific conclusions of the article in any way. The original article has been updated.

